# MyD88 Plays an Important Role in UVB-Induced Suppression of Dendritic Cell Activity, T Cell Function, and Cutaneous Immune Response

**DOI:** 10.3390/ijms26199361

**Published:** 2025-09-25

**Authors:** Mohammad Asif Sherwani, Carlos Alberto Mier Aguilar, Charlotte McRae, Gelare Ghajar-Rahimi, Aisha Anwaar, Ahmed Omar Jasser, Ariq Chandra, Hui Xu, Nabiha Yusuf

**Affiliations:** 1 Department of Dermatology, University of Alabama at Birmingham, 1670 University Boulevard, VH 566A, Birmingham, AL 35294-0019, USA; 2 Heersink School of Medicine, University of Alabama at Birmingham, Birmingham, AL 35233, USA; 3 O’Neal Comprehensive Cancer Center, University of Alabama at Birmingham, Birmingham, AL 35233, USA

**Keywords:** ultraviolet radiation, DNA damage, immune suppression, MyD88, TRIF

## Abstract

Ultraviolet B (UVB) radiation triggers DNA damage and immune suppression, establishing conditions favorable for skin carcinogenesis. Previous studies have shown that a downstream adaptor for Toll-like receptors (TLRs), myeloid differentiation primary response 88 (MyD88), plays a role in UVB-induced DNA damage and immunosuppression. However, specific mechanisms for the effects on dendritic cells and T cells remain poorly understood. The objective of this study is to determine the role of MyD88 and TIR-domain-containing adaptor inducing interferon-β (TRIF), another key TLR downstream adaptor, in UVB-induced suppression of dendritic cell activity and T cell function. *MyD88−/−*, *Trif−/−*, and wild-type (WT) mice were evaluated for UVB-induced effects on dendritic cell, T cells, and contact hypersensitivity responses in skin. *MyD88−/−* mice exhibited significant resistance to UVB-induced immune suppression, compared to *Trif−/−* mice and wild-type controls. The *MyD88* deficiency significantly reduced UVB-induced Treg cells that were CD4^+^CD25^+^Foxp3^+^ and produced interleukin (IL)-10. Moreover, it significantly inhibited the UVB-induced suppression of IL-12/IL-23 producing CD11c^+^ dendritic cells. Further experiments confirmed that MyD88 conditional knockout (*MyD88fl/flXCD11c.Cre*) mice were protected against UVB-induced immune suppression. Dendritic cells from MyD88 genomic or conditional knockout mice were resistant to UVB-induced reduction of major histocompatibility complex (MHC) class II antigens. These findings show that MyD88 plays a key role in UVB-induced immune suppression. The deficiency in the MyD88 gene inhibits UVB-induced suppression of CD11c+ dendritic cell (DC) activity and reduces UVB-induced development of Treg cells. Our studies demonstrate a new mechanism for MyD88-mediated regulation of UVB-induced immune suppression.

## 1. Introduction

Ultraviolet B (UVB) radiation (290–320 nm) initiates a cascade of cellular events leading to DNA damage, immune suppression, and non-melanoma skin cancers [1,2,3]. The clinical significance of UVB-induced pathology is demonstrated through multiple lines of evidence. Immunocompromised solid organ transplant recipients have an elevated risk of skin cancer, with a 3-, 10-, and 65-fold increased risk for developing malignant melanoma, basal cell carcinoma, and cutaneous squamous cell carcinoma [4]. Additionally, experimental studies show that even sub-carcinogenic doses of UV radiation suppress immune responses sufficiently to permit the outgrowth of highly antigenic UV-induced skin tumors [2,3]. Finally, in humans, UV-induced suppression shows genetic determination, and individuals with UV-suppressed cell-mediated immunity demonstrate increased skin cancer risk [2], exhibiting the direct connection between immune function and carcinogenesis.

The molecular pathogenesis of UVB damage centers on cyclobutane pyrimidine dimer (CPD) formation and subsequent cellular responses [5]. Beyond direct DNA damage, CPDs trigger immune dysfunction by altering antigen-presenting cell (APC) behavior, representing one of the earliest molecular events in UVB-induced immune suppression [6]. UV-irradiated dendritic cells exhibit impaired migration from skin-draining lymph nodes and reduced capacity to stimulate helper and effector T-cells [6,7]. This immune suppression involves elevated interleukin (IL)-10 production and regulatory T-cell activation [8,9], establishing conditions favorable for tumor development.

The innate immune system, particularly Toll-like receptor-4 (TLR4), plays a central role in mediating UVB-induced cellular damage and immune suppression [10,11]. TLR4 and its downstream effector myeloid differentiation primary response 88 (MyD88) are expressed in human skin tumors [12], where their signaling inhibits DNA repair and promotes immune suppression [10,11,13]. This pathway functions through MyD88-dependent recruitment of interleukin-1 receptor-associated kinase-4 (IRAK4 and IRAK1), leading to pro-inflammatory mediator production [12]. The DNA repair cytokines IL-12 and IL-23, produced by APCs, stimulate DNA repair gene expression and reduce CPDs through NER pathways [14,15]. Specifically, IL-12 plays a crucial role in promoting T-helper 1 responses and preventing UV-induced immunosuppression by breaking immunotolerance, antagonizing T regulatory cells, and promoting expression of DNA repair enzymes [14,16,17,18].

While the role of TLR4/MyD88 signaling in UV-induced immunosuppression is established, the specific mechanisms by which MyD88 regulates UVB-induced effects on dendritic cells and T cells remain undefined. This study investigates how MyD88 deficiency affects UVB-induced suppression of dendritic cell activity and development of Treg cells following UVB exposure. By utilizing genetic and conditional MyD88 knockout models, we examine how MyD88-signaling affects UVB-induced immune suppression through the regulation of dendritic cells and Treg cells. Understanding these cellular and molecular pathways will advance therapeutic strategies for preventing UVB-induced immune suppression in at-risk populations.

## 2. Results

### 2.1. The Deficiency in the MyD88 Gene Diminishes UVB-Induced Suppression of Cutaneous Immune Responses

**Figure 1:**
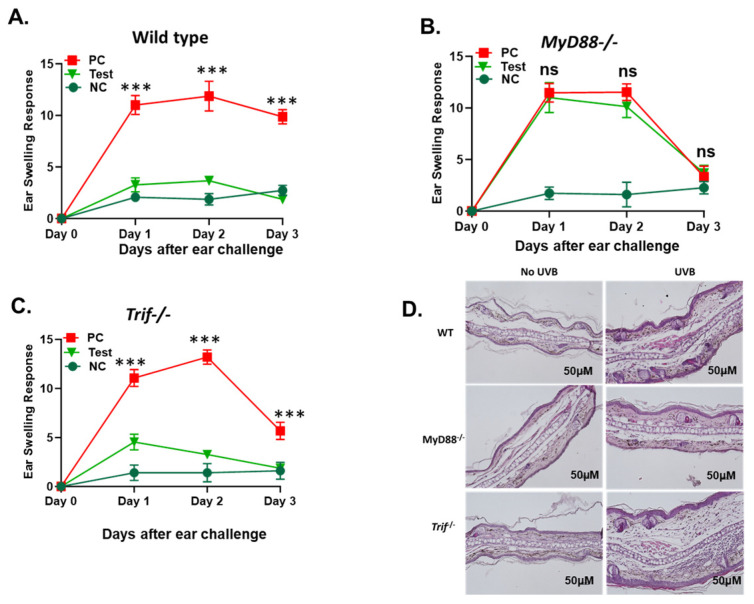
The loss of TRIF does not interfere with UVB-induced suppression of contact hypersensitivity. (**A**) WT, (**B**) *MyD88−/−*, and (**C**) *Trif−/−* mice were subjected to the DNFB model of hypersensitivity as outlined in the Methods. Test group mice (UVB) were subjected to UVB pre-treatment (200 mJ/cm^2^) on their dorsal side for 4 days, sensitized with 50 µL of 0.5% 2,4-dinitrofluorobenzene (DNFB; 4:1::acetone:olive oil) on the UVB treated site 24 h later, then re-challenged with 20 µL of 0.2% DNFB on their ears 5 days after sensitization. Positive control (PC) mice were sensitized with DNFB on the dorsal skin and re-exposed to DNFB on the ear. Negative control (NC) mice were only exposed to DNFB on the ear. The degree of hypersensitivity response was measured via change in auricular thickness ± SD for 3 days following re-exposure to DNFB. (*** *p* < 0.001; ns = not significant) (**D**) Representative hematoxylin and eosin (H&E) staining of ear skin samples collected 24 h post exposure. Scale bar = 50 μM.

UVB is a major risk factor for UVB-induced immune suppression [19]. We have previously shown that mice that are deficient in TLR4 are protected from UVB-induced immune suppression [11]. TLR4 mediates its effect via MyD88 and TIR-domain-containing adaptor inducing interferon-β (TRIF) pathways [20]. The TLR4/MyD88 axis has been reported to cause immune suppression in mice [13]. TLR4 also signals via TRIF pathway from endosomes, the latter activating interferon regulatory factor-3 (IRF3) and driving type I interferon (IFN) production. Since type I IFNs can enhance UV-induced DNA repair and modulate photoimmunity, we hypothesized that TRIF-dependent signaling might counterbalance MyD88-mediated immunosuppressive effects downstream of TLR4. Therefore, we directly compared MyD88 and TRIF using global knockouts [21,22,23,24]. To determine the respective roles of MyD88 and TRIF pathways, *MyD88−/−*, *Trif−/−* mice were compared to wild type (WT) mice following UVB-induced immune suppression [19]. In this protocol, ear swelling caused by edema represents a robust contact hypersensitivity response (Figure 1A–C, PC groups) and successful UVB-induced immune suppression mitigates ear swelling (Figure 1A–C). There was significantly more (*** *p* < 0.001) UVB-induced immunosuppression in *Trif−/−* and WT mice compared to *MyD88−/−* mice. *MyD88−/−* mice were resistant to UVB-induced immune suppression. Histologic examination of ears of these mice similarly revealed extensive edema in *MyD88−/−* mice compared to *Trif−/−* and WT mice following DNFB exposure in the setting of UVB pre-treatment [Figure 1D].

### 2.2. The MyD88 Deficiency Inhibits UVB-Induced Development of T Regulatory Cells

**Figure 2:**
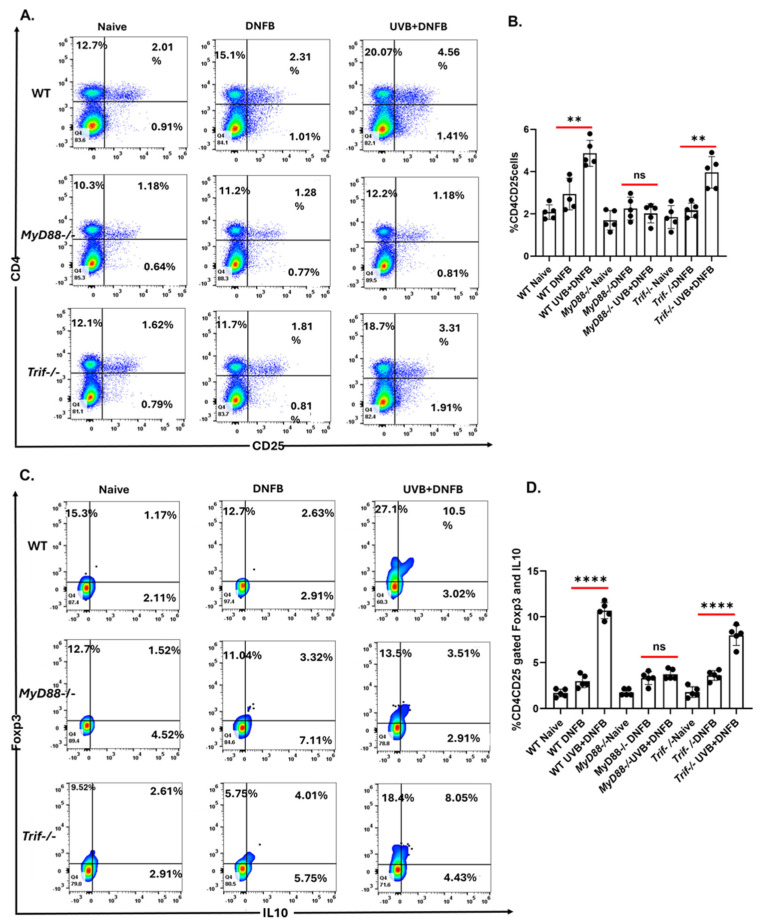
MyD88 deficiency suppresses the development of UVB-induced regulatory T-cells. *MyD88−/−*, *Trif−/−*, and WT mice were subjected to 4 days of UV exposure (200 mJ/cm^2^) and 24 h later they were sensitized with 50 µL of 0.5% DNFB. Five days after sensitization, mice were sacrificed, and lymph nodes were collected for analysis by flow cytometry. (**A**) Representative flow cytometry plots of CD4 and CD25 populations. (**B**) Expression of double positive (CD4^+^CD25^+^) cells. (**C**) Representative flow cytometry plots of Foxp3 and IL-10 expression within the CD4^+^CD25^+^ population. (**D**) Percentage of CD4^+^CD25^+^ double positive cells expressing Foxp3 and IL-10. **** *p* < 0.0001; ** *p* < 0.01; ns = not significant; *n* = 5 per group, and each experiment was repeated twice.

UVB induces the development of CD4^+^CD25^+^Foxp3^+^ regulatory T-cells that secrete IL-10 [25]. UVB-induced regulatory T-cells play an important role during immunosuppression and photocarcinogenesis, due to their ability to inhibit anti-tumoral effector functions [26,27,28,29]. CD4^+^CD25^+^Foxp3^+^ T-cells can be found intermingled with human basal cell carcinoma along with the T-helper 2 cytokines IL-4 and IL-10 [30]. To determine whether MyD88 or TRIF contributed to the generation of T regulatory cells, *MyD88−/−*, *Trif−/−*, and WT mice were subjected to a local UVB immunosuppression regimen as mentioned in the Methods section. Mice were sacrificed after DNFB treatment, and single-cell suspensions of cells were prepared from lymph nodes. In the setting of UVB pretreatment, the prevalence of CD4^+^CD25^+^ regulatory T-cells increases in WT mice following DNFB exposure. *Trif−/−* mice exhibited a similar but blunted increase in CD4^+^CD25^+^ regulatory T-cells in this condition. *MyD88−/−* mice developed significantly fewer CD4^+^CD25^+^ regulatory T-cells than WT and *Trif−/−* mice [Figure 2A,B]. Amongst the regulatory T-cells found present in the UVB + DNFB condition, a significantly larger proportion (*** *p* < 0.001) were Foxp3^+^IL10^+^ in WT and *Trif−/−* compared to *MyD88−/−* mice [Figure 2C,D].

### 2.3. The MyD88 Deficiency Inhibits UVB-Induced Reduction of IL12p39 and IL23p19 Production by CD11c+ DC

**Figure 3:**
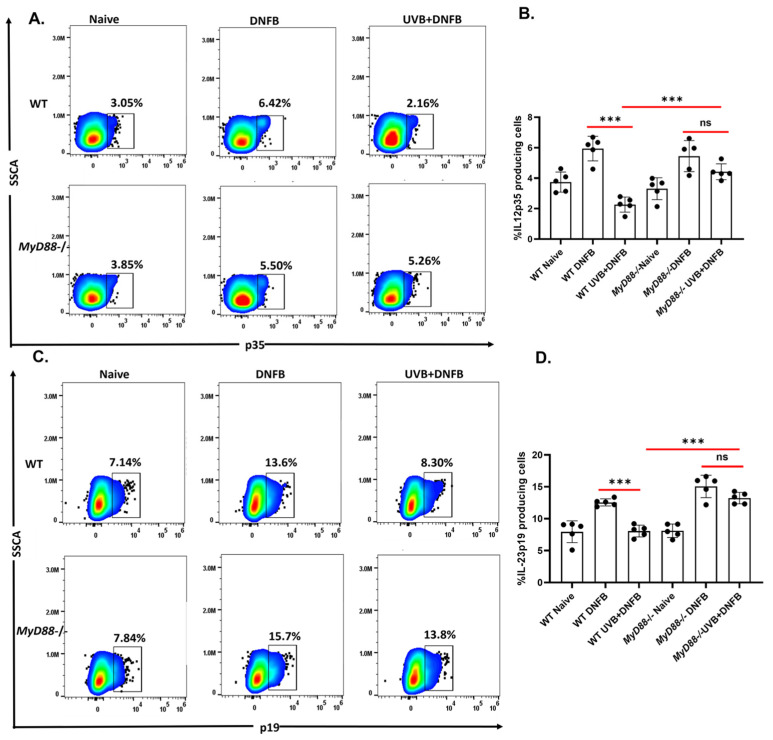
The MyD88 deficiency diminishes UVB-induced suppression of IL-12p35 and IL-23p19 by CD11c+ dendritic cells. *MyD88−/−* and WT mice were subjected to 4 days of UV exposure (200 mJ/cm^2^) and 24 h later they were sensitized with 50 µL of 0.5% DNFB. Five days after sensitization, mice were sacrificed, and lymph nodes were collected, and single cell suspension was prepared. CD11c+ cells were identified by flow cytometry and intracellular staining was performed for cytokine IL-12p35 (**A**,**B**) and IL-23p19 (**C**,**D**) by flow cytometry. *** *p* < 0.001; ns = not significant; *n* = 5 per group and each experiment was repeated twice.

The cytokines IL-12 and IL-23 are heterodimers, sharing a common p40 (beta chain) subunit that is combined with either a p35 alpha chain (IL-12) or p19 alpha chain (IL-23). IL-12 and IL-23 promote cell-mediated responses driven by different subsets of T-helper cells [31,32,33]. Both IL-12 and IL-23 have been reported to stimulate XPA to initiate repair of CPDs [14,15]. We have previously reported that these cytokines were upregulated in the absence of TLR4 [10]. To determine whether protection against immune suppression observed in the absence of MyD88 is mediated by IL-12 and IL-23, *MyD88−/−* and WT mice were subjected to UVB-induced immune suppression protocol then exposed to DNFB. Five days after sensitization, mice were sacrificed, and lymph node cells were stained for Il-12p35 in CD11c^+^ dendritic cells by intracellular staining and quantified by flow cytometry. Expression of IL-12p35 was significantly higher (*** *p* < 0.001) in CD11c^+^ dendritic cells from *MyD88−/−* mice than those in WT mice [Figure 3A,B]. Similar results were observed for IL-23p19, which was significantly higher (*** *p* < 0.001) in CD11c^+^ dendritic cells from *MyD88−/−* mice than in WT mice [Figure 3C,D].

### 2.4. The Conditional Knockout of the MyD88 Gene in CD11c+ DC Prevents UVB-Induced Immunosuppression

**Figure 4:**
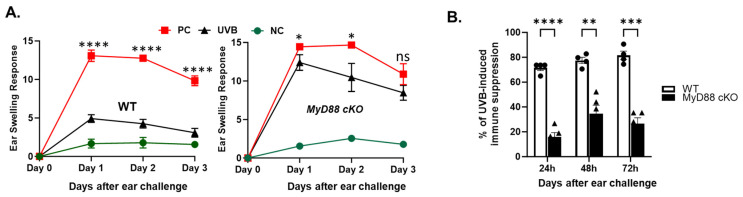
The conditional knockout of the MyD88 gene in CD11c+ cells significantly inhibits UVB-induced immune suppression. (**A**) *MyD88 fl/fl* (WT) and *MyD88 fl/fl*.CD11c.Cre (*MyD88* cKO) mice were subjected to the DNFB model of hypersensitivity. Test group mice were subjected to UVB pre-treatment (200 mJ/cm^2^) on their dorsal side for 4 days, sensitized with 50 µL of 0.5% 2,4-dinitrofluorobenzene on the UVB treated site 24 h later, then re-challenged with 20 µL of 0.2% DNFB on their ears 5 days after sensitization. Positive control (PC) mice were sensitized with DNFB on the dorsal skin and re-exposed to DNFB on the ear. Negative control (NC) mice were only challenged to DNFB on the ear. The degree of hypersensitivity response was measured via change in auricular thickness ± SD for 3 days following re-exposure to DNFB. (**B**). Further analysis evaluated the percentage of UVB-induced immune suppression compared to PC and NC in WT and *MyD88* cKO mice with the following equation: ${\mathrm{IS}}_{\mathrm{UVB}}=100(1-\frac{x_{UVB}-\bar{NC}}{\bar{PC}-\bar{NC}})$ for each time point. Data are presented as mean ± SE (*n* = 3–5). Two-way ANOVA/mixed-effects model with post hoc Tukey’s multiple comparisons test (**** *p* < 0.0001, *** *p* < 0.001, ** *p* < 0.01, * *p* < 0.05, ns = not significant).

UV-irradiated antigen-presenting dendritic cells have a reduced ability to stimulate helper and effector T-cells [34,35]. To further confirm the role of dendritic cells in UVB-induced immune suppression, *MyD88* cKO (*MyD88fl/fl.CD11c.Cre*) and WT mice were subjected to UVB-induced immune suppression protocol as mentioned earlier. There was significantly less UVB-induced immunosuppression in *MyD88* cKO mice (22%, 37%, and 45%) compared to WT mice (78%, 79%, and 67%) at 24 h, 48 h, and 72 h, respectively [Figure 4A,B].

**Figure 5:**
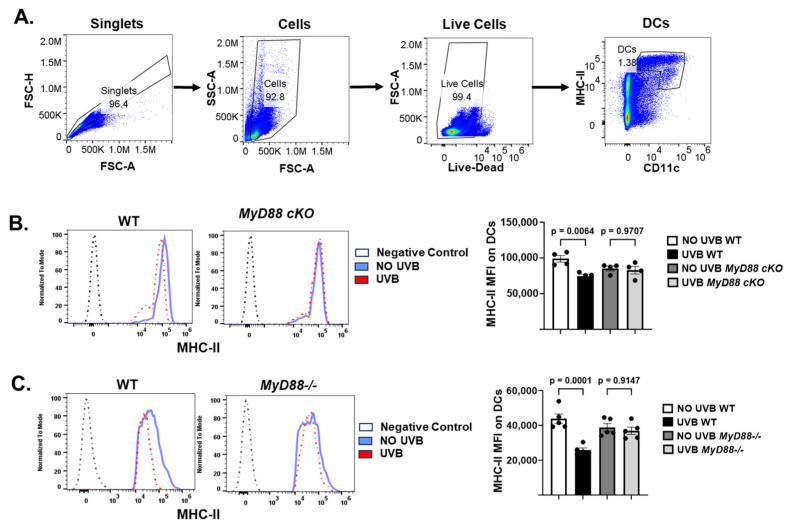
MyD88 deficiency inhibits UVB-induced decrease of MHC class II antigens by dendritic cells. Panels of (**A**) mice were exposed to UVB for 4 consecutive days. On day 5, the draining lymph node cells were collected for analysis by flow cytometry. (**A**) CD11c/MHC class II positive dendritic cells (DCs) were gated. Median fluorescence intensity (MFI) of MHC-II was evaluated. (**B**) MHC-II expression level by WT and MyD88 cKO DCs before and after UVB radiation (*n* = 4). (**C**) MHC-II expression level by WT and *MyD88−/−* DCs before and after UVB radiation (*n* = 5). Data are presented as mean ± SEM, One-Way ANOVA with post hoc Tukey’s multiple comparisons test was used for statistical analysis.

Further tests showed that dendritic cells from the draining lymph nodes of *MyD88−*/− or *MyD88*-cKO mice were resistant to UVB-induced reduction of MHC class II antigen expression, compared to dendritic cells from UVB-treated wild-type mice (Figure 5A–C). The results support the role of MyD88 signaling in UVB-induced suppression of dendritic cell activity.

## 3. Discussion and Conclusions

This study investigates the differential roles of the TLR4 downstream adaptors, MyD88 and TRIF, in mediating UVB-induced cutaneous immune responses. While TLR4 signaling blockade has been previously shown to enhance in DNA damage repair [10,36], augment anti-tumor immune activation [11], and reduce risk of skin carcinogenesis [36,37], the contributions of two key TLR4 downstream adaptors, MyD88 and TRIF, remained less well understood. Our results reveal an important role of MyD88 in UVB-induced suppression of DC activity and development of Treg cells. The outcome advances the understanding of the effect of MyD88 in UVB-induced immunosuppression.

The increased production of IL-12p35 and IL-23p19 by dendritic cells in *MyD88−/−* mice provides an additional mechanistic link between MyD88 signaling and UVB-induced immune suppression [14,15]. IL-12p35 and IL-23p19 expression by CD11c^+^ cells is comparable between *MyD88−/−* and WT mice in naïve conditions.

UVB exposure induces immunosuppression, a well-documented phenomenon that contributes to the development of skin cancers by impairing immune responses to tumor antigens. To investigate how MyD88 and TRIF might modulate immune responses following UVB exposure, we next turned to the DNFB model of contact hypersensitivity. In this model, ear edema serves as an indicator of the general inflammatory response upon re-challenge with DNFB. As expected, in WT mice re-exposed to DNFB after sensitization an immune response is mounted and ear edema ensures. However, pre-treating with UVB suppresses the immune response and significantly blunts hypersensitivity response (Figure 1A PC vs. UVB Groups).

*MyD88−/−* mice were significantly resistant to UVB-induced immune suppression unlike *Trif−/−* and WT controls in which UVB pretreatment (Figure 1A,C UVB Groups) exhibited reduced ear swelling and histological signs of edema after sensitization with DNFB compared to PC groups. By day 3 following re-challenge with DNFB, ear edema in *MyD88−/−* mice was statistically similar to negative control groups irrespective of UVB-pretreatment (Figure 1B, PC, and UVB vs. NC groups), suggesting that MyD88 deletion can modulate hypersensitivity response independent of UVB treatment. Interestingly, the ear edema in *Trif−/−* mice did subjectively appear to resolve more quickly than WT mice; however, temporal dynamics were not the primary focus of this study.

Additionally, our experiments suggest that the resistance to UVB-induced immune suppression in *MyD88−/−* mice is partly due to CD11c^+^ cells. When we performed hypersensitivity tests in *MyD88 fl/fl.CD11c.Cre* (*MyD88* cKO) mice, where MyD88 is conditionally deleted in the CD11c^+^ compartment, UVB-induced suppression of DNFB-contact hypersensitivity was significantly diminished compared to WT mice, although not completely as observed in the global *MyD88−/−* mice. It suggests that MyD88 signaling in other cells may have a role in UVB-induced immune suppression. While direct comparison of *MyD88* cKO and global *MyD88−/−* mice was beyond the scope of this study, these experiments nevertheless underscore the central role of dendritic cells in mediating MyD88-dependent effects.

A mechanism for the resistance to UVB-induced immune suppression in *MyD88−/−* mice is reduction in regulatory T-cell populations in the draining lymph nodes after UVB exposure. CD4^+^CD25^+^IL-10^+^ regulatory T-cells are key mediators of UVB-induced immune suppression [29] and promote tumor progression by shutting down immune responses. *MyD88−/−* mice exhibited reduced regulatory T-cell populations post UVB exposure, preserving immune function. In contrast to *MyD88−/−*, *Trif−/−* mice, like WT mice, accumulated Foxp3^+^IL-10^+^ regulatory T-cells following DNFB exposure in the setting of UVB pretreatment, suggesting that TRIF signaling plays a lesser, or possibly counter-regulatory, role in UVB-induced immune suppression.

Dendritic cells play an important role in the development of regulatory T cells. UVB inhibits dendritic cell activity and induces tolerogenic dendritic cells for the development of regulatory T cells. CD11c^+^ dendritic cells in skin draining lymph nodes of *MyD88−/−* mice retained the ability to produce cytokines like IL-12 and IL-23, maintaining their ability to stimulate helper and effector T-cells. Moreover, further analysis shows that dendritic cells from *MyD88−/−* or *MyD88*-cKO mice are resistant to UVB-induced decrease of MHC class II antigens. It supports that the activity of dendritic cells is protected from UVB by deficient MyD88 signaling. Taken together, these experiments suggest that UVB exposure impairs dendritic cell function and promotes regulatory T-cell formation through MyD88-dependent pathways.

In addition to canonical inflammatory outputs, UVR can engage TLR4 as a death receptor via an IRAK-independent, FADD-dependent pathway in macrophages, which may contribute to photoimmunosuppression and cell-type specific responses [38]; this mechanism complements our observation that MyD88 signaling in CD11c+ cells modulates UVB-induced immune suppression.

Based on the results of this study, we postulate that targeting MyD88 signaling holds significant promise as a therapeutic strategy to mitigate the harmful effects of UV exposure, particularly in individuals at higher risk of skin cancer such as those in immunocompromised states or with history of chronic UV exposure. This work lays the foundation for future studies employing MyD88 inhibitors (e.g., ST2528 [39] or M20 [40]) in skin carcinogenesis models. As with any immune modulatory therapy, optimal intervention depends heavily on the desired clinical outcome. While MyD88 inhibition may be beneficial for reducing tumorigenesis, it could also increase the risk of excessive immune activation and hypersensitivity. In conclusion, this study provides evidence for the roles of MyD88 and TRIF in the TLR4-mediated effects of UVB-induced immune suppression, and lays the groundwork for future studies of the intricate interplay between MyD88, TLR4, and the immune milieu.

## 4. Materials and Methods

### 4.1. Animals and Reagents

*MyD88−/−* (stock #009088), *MyD88fl/fl* (stock #008888), *Trif−/−* (stock #005037), and *CD11c.Cre* (stock# 008068) male and female mice on a C57BL/6 background were purchased from The Jackson Laboratories (Bar Harbor, ME, USA). All procedures were approved by the Institutional Animal Care and Use Committee (IACUC) before the initiation of any studies (IACUC-22314). To ensure rigor and reproducibility, we used 5 age-matched mice per panel. Both male and female mice were included in approximately equal numbers (*n* = 3–5 per sex per group), and all the experiments were repeated twice for reproducibility. We did not observe consistent sex-related differences in CHS or UVB-induced suppression; therefore, data from males and females were pooled.

*MyD88fl/fl* mice were crossed with CD11c-Cre mice to generate *MyD88fl/fl* × CD11c-Cre (*MyD88* cKO) and Cre-negative littermate controls on a C57BL/6 background. Both sexes, 8–12 weeks old, were used. Genotyping was performed per JAX protocols; Cre expression is restricted predominantly to conventional dendritic cells.

### 4.2. Antibodies and Reagents

Flow cytometry studies employed the following antibodies: anti-mouse CD16/CD32 (2.4G2; Purified), CD4 (RM4-5; AF700, PerCP-Cy5.5 and GK1.5; AF488), CD11c (N418; AF488, AF700, PE), CD25 (PC61.5; APC, AF700), Foxp3 (FJK-16s; PE), IL-10 (JES5-16E3; PerCp), MHC Class II (I-A/I-E, M5/114.15.2: SB 436), IL-12 (27537; PE, PerCP), and IL-23 (fc23cpg; AF488) were from Thermofisher Scientific (Waltham, MA, USA). 2,4-dinitrofluorobenzene (DNFB) was purchased from Millipore-Sigma (St. Louis, MO, USA).

### 4.3. Contact Hypersensitivity

The induction and elicitation of contact hypersensitivity (CHS) in mice were carried out as previously described [41], with additional clarifications provided below. Briefly, both male and female mice (*n* = 3–5 per sex per group; total group size = 6–10) were used. No consistent sex-related differences in ear swelling or UVB-induced suppression were observed; therefore, data were pooled. Mice were sensitized on the shaved and depilated abdominal skin using 50 μL of 0.5% 2,4-dinitrofluorobenzene (DNFB, dissolved in 4:1 acetone:olive oil). For depilation, Nair™ depilatory cream (Church & Dwight Co., Ewing, NJ, USA) was applied to the abdominal area 24 h prior to sensitization. Five days later, mice were challenged on the dorsal side of both ear pinnae with 20 μL of 0.2% DNFB. Ear thickness was measured using a digital micrometer (Mitutoyo, Kawasaki, Japan) at baseline and daily for 3 consecutive days following the challenge. For histology, ear tissue samples were collected 24 h after DNFB challenge and processed for hematoxylin and eosin (H&E) staining. In experimental groups, mice were pre-exposed to UVB (200 mJ/cm^2^, dorsal skin) once daily for 4 consecutive days prior to DNFB sensitization. Control groups included: (i) positive control (PC; sensitized and challenged without UVB), (ii) negative control (NC; challenged only), and (iii) UVB + DNFB groups as described above (Supplementary Figure S1).

### 4.4. Flow Cytometry Analysis

Following the contact hypersensitivity procedure, draining lymph nodes were harvested from panels of *MyD88−/−*, *Trif−/−*, and WT mice, to make single cell suspensions [41]. Cells were stained with fluorescence-labeled antibodies against CD4, Foxp3, CD25, and intracellular cytokines IL-10. To examine dendritic cells, the draining lymph node cells were harvested 24 h after the last UVB exposure and stained with antibodies to CD11c, IA/IE, IL-12, and IL-23. All the cell populations were analyzed in a flow cytometer (Attune NxT, Thermofisher Scientific, Waltham MA, USA) as previously described [41] and the analysis was performed using FlowJo software version 10.6.0.

### 4.5. Statistical Analysis

In all experiments, UVB-exposed and -unexposed groups were compared separately using one-way analysis of variance (ANOVA). For ear swelling responses, negative control and UVB groups were individually compared with positive controls. All quantitative data are shown as the means ± SD. Unless otherwise stated in figure legends, *p* < 0.05 was considered statistically significant.

## Data Availability

The data presented in this study are available on request from the corresponding author.

## Supplementary Materials

The supporting information can be downloaded at https://www.mdpi.com/article/10.3390/ijms26199361/s1.
