# Supplementary material for: MyD88 Plays an Important Role in UVB-Induced Suppression of Dendritic Cell Activity, T Cell Function, and Cutaneous Immune Response
Source: Int J Mol Sci. 2025 Sep 25;26(19):9361. doi: 10.3390/ijms26199361 (PMC12524367; doi:10.3390/ijms26199361)
Supplement: Supplementary file 1 [file ijms-26-09361-s001.zip › ijms-3845909-supplementary.pdf]

UVB INDUCED IMMUNOSUPRESSION PROTOCOL

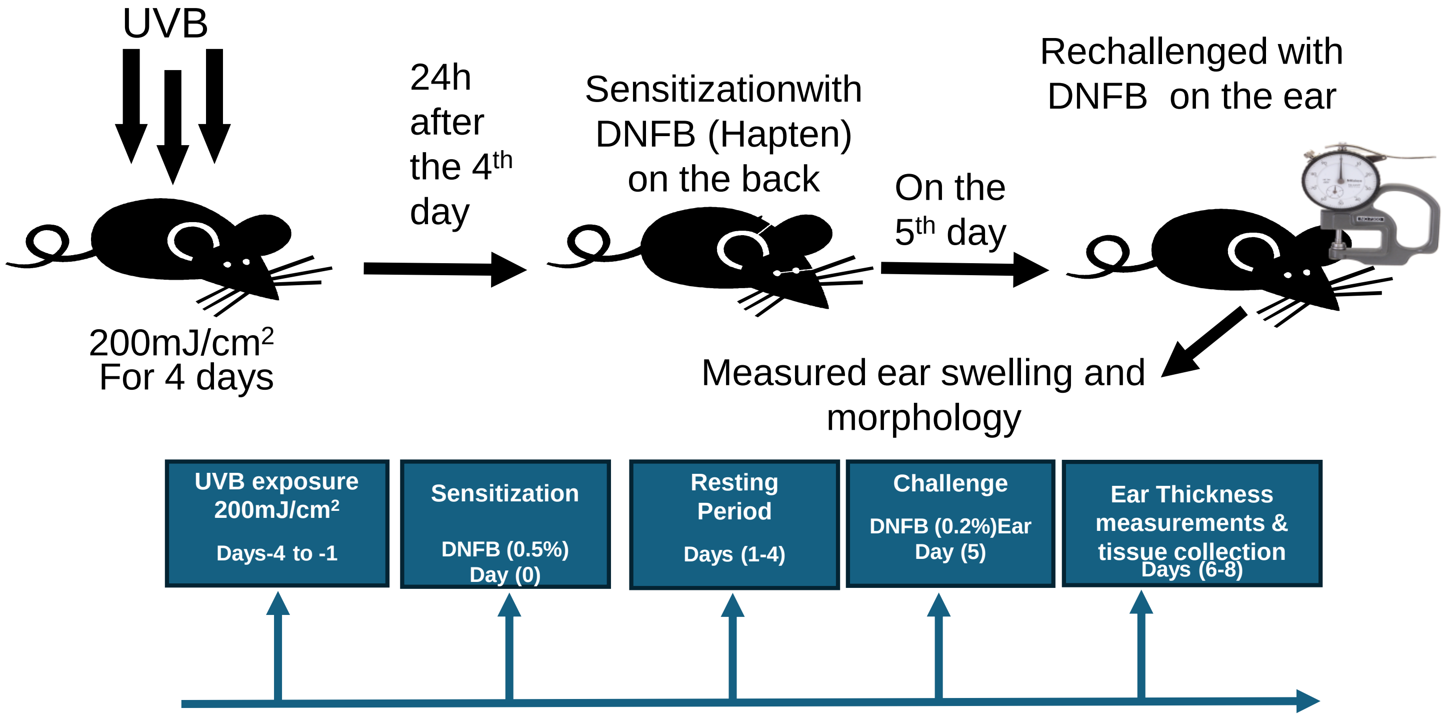

Animal	Group	UVB	DNFB	DNFB (ear)
WT	NC	-	-	+
	PC	-	+	+
	UVB	+	+	+
MyD88-/-or Trif-/-	NC	-	-	+
	PC	-	+	+
	UVB	+	+	+

Figure.S1. Schematic of experimental protocol showing UVB pre-treatment, DNFB sensitization, challenge, and endpoint analyses

Figure S1. Schematic of experimental design for UVB-induced suppression of contact hypersensitivity. Wild-type (WT), MyD88-/-, and Trif-/- mice were exposed to UVB radiation (200 mJ/cm^2, once daily for 4 consecutive days; Days –4 to –1). On Day 0, mice were sensitized with 50 μL of 0.5% DNFB on the abdomen. After a 5-day resting period (Days 1–4), mice were challenged with 20 μL of 0.2% DNFB on the ear pinnae (Day 5). Ear thickness was measured daily for 3 days, and ear tissue and draining lymph nodes were collected for histological and immunological analyses (Days 6–8)
